# Assessing tumor molecular profiling to guide treatments for patients with advanced female genital tract malignancy

**DOI:** 10.18632/oncotarget.23675

**Published:** 2017-12-27

**Authors:** Philip Carter, Costi Alifrangis, Biancastella Cereser, Pramodh Chandrasinghe, Lisa Del Bel Belluz, Christina Fotopoulou, Andreja Frilling, Thomas Herzog, Nina Moderau, Neha Tabassum, Jonathan Krell, Justin Stebbing

**Affiliations:** ^1^Department of Surgery and Cancer, Imperial College, London, UK; ^2^Department of Oncology, University College Hospital, London, UK; ^3^Department of Surgery, University of Kelaniya, Kelaniya, Sri Lanka; ^4^Department of Obstetrics and Gynecology, University of Cincinnati, Cincinnati, USA; ^5^University of Cincinnati Cancer Institute, University of Cincinnati, Cincinnati, USA

**Keywords:** tumor profiling, female genital tract malignancy, cancer treatment

## Abstract

Tumor molecular profiling has enabled selection of targeted therapies in a host of solid tumors. Here we used a retrospective clinical cohort, to evaluate the benefit of tailoring treatments for female genital tract malignancy, using tumor molecular profiles. Clinical outcome data for 112 patients was retrospectively separated into two groups. These either followed a matched treatment plan that incorporated at least one drug recommended according to their tumor profile and none that were expected to have no benefit (64 patients), or was unmatched with suggested treatments and received at least one drug that was anticipated to lack benefit for that tumor (48 patients).

In the group of patients whose drugs matched those recommended by molecular profiling of their tumor, their overall survival was 593 days on average, compared to 449 days for patients that did not; removing drugs predicted to have no benefit from treatment regimens received after profiling increased survival by 144 days on average (*P* = 0.0265). In the matched treatment group, 30% of patients had died by the last time of monitoring, whereas this was 40% in the unmatched group (*P* = 0.2778). The IHC biomarker for the progesterone receptor was demonstrated to be prognostic for survival.

## INTRODUCTION

Cancers of the female reproductive system including cervical cancer, cancer of the uterus, ovary, vulva, vagina, and fallopian tube cause significant numbers of cancer deaths and morbidity worldwide. Cancer of the female genital tract is the third most frequently occurring type of malignancy in women [1].

Excluding high grade serous ovarian cancers, the clinical management of the gynaecological malignancies mentioned above remains reliant on surgery in early or localized disease, with radiotherapy and chemotherapy utilizing platinum-based regimens in locally advanced or metastatic disease [2–4]. The overexpression of the estrogen receptor (ER) and/or progesterone receptor (PR) in endometrial cancer has been effectively utilized in therapeutic strategies in metastatic disease [5–7]. However many cancers such as uterine carcinosarcoma, uterine and ovarian clear cell carcinoma, and other histologies, are typically lacking in targeted treatment options, despite recent data that indicates benefit in blocking the HIF-1/VEGF signaling pathway [8].

Here we used data from Caris Life Sciences to find if tumor molecular profiling led to better patient outcomes, and the impact on drug usage.

## RESULTS

**Figure 1:**
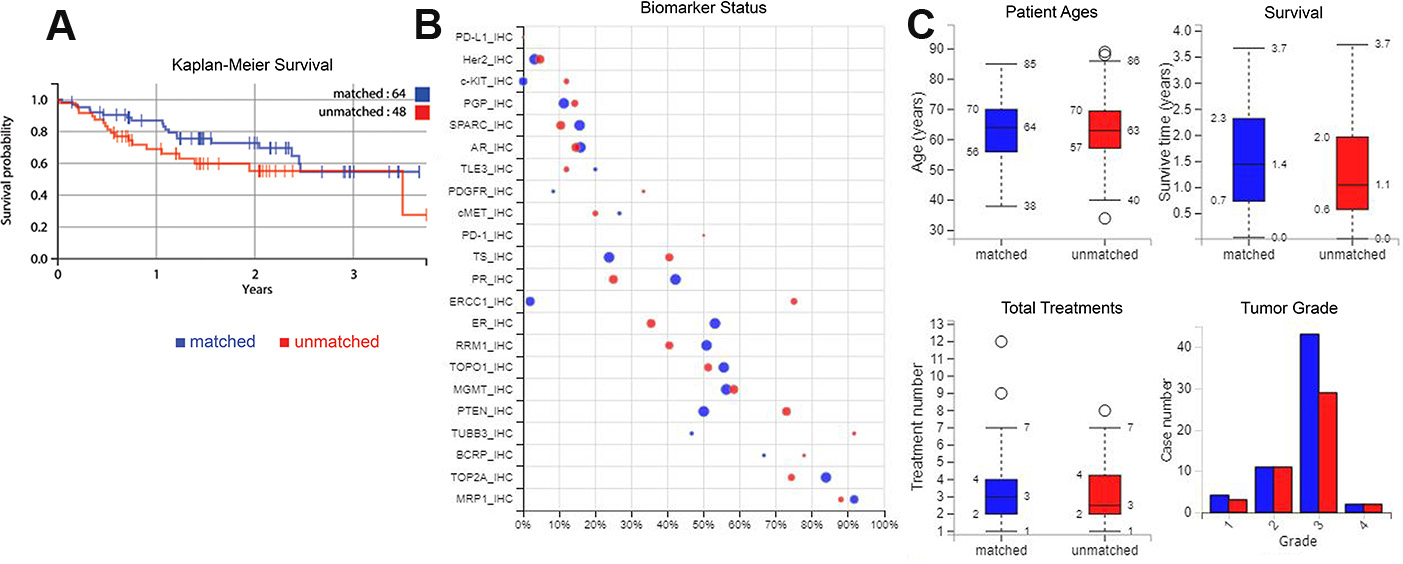
Survival, biomarker, patient age, treatment number and tumour grade characteristics (**A**) Kaplan-Meier curve of the increase in overall survival for patients treated only with therapies profiled to be beneficial compared to patients who had one or more therapies predicted to have no benefit. (**B**) Biomarkers compared for tumors from the matched and unmatched patients; positive ratio gives the percentage that have positive biomarker results i.e. for IHC, positive is protein expression above a certain threshold, and for sequencing biomarkers, positive is a gene mutation that is generally expected to be pathogenic. The size of a circle shows the number of cases. (**C**) Age, treatment numbers, survival time and grade of samples compared between the two treatment types. Blue = matched, red = unmatched.

Clinical data describing treatments and outcomes for 112 advanced stage female genital tract cancer patients from Caris Life Sciences was analyzed retrospectively; the patients were treated in a number of clinics in the USA. Baseline information regarding histology, staging, age and previous treatment was noted at time of Caris profiling (Tables 1 and 2, Figure 1C–see plots on the right-hand side). Survival from point of testing was ascertained. Patients’ treatment was classified as matched or unmatched. This depended on whether the drugs selected by the clinician following the date of collection for profiling were predicted to be beneficial from analysis of the molecular profile of the tumor. As shown in Table 2, endometrial cancer is the most prevalent type within this cohort (25%).

**Table 1:** Ages of patients in both treatment groups

Age	Matched	Unmatched
20–29	0	0
30–39	3	1
40–49	7	7
50–59	13	8
60–69	24	20
70–79	15	6
80–89	2	6

**Table 2:** Patient and tumor information for the matched and unmatched groups compared with all

Group	Patient & Tumor Information						
	Age	Ethnicity	Histology	Grade	Stage	Survival (Days)	Mortality %
All patients (112)	62.7	White: 92	Endometrioid adenocarcinoma, NOS: 28	Grade 4/ Undifferentiated: 4 (4%)	IV: 28 (25%)	531	34
		Black/African American:15	Squamous cell carcinoma, NOS: 11	Grade 3/ Poorly differentiated: 72 (64%)	III no IIIC: 22 (19%)		
		Asian: 2	Mixed cell adenocarcinoma: 10	Grade 2 / Moderately differentiated: 22 (20%)	IIIC: 16 (14%)		
		Other/unknown: 1	Serous cystadenocarcinoma, NOS: 9	Grade 1 / Well differentiated: 7 (6%)	II: 12 (11%)		
		Hawaiian/Pacific Islander: 1	Adenocarcinoma, NOS: 9	Unknown / Not determined: 6 (5%)	I: 31 (28%)		
		American Indian/Alaskan Native: 1	Carcinosarcoma, NOS: 8	None / Not applicable: 1 (1%)	Unknown: 3 (3%)		
			Papillary serous cystadenocarcinoma: 5				
			Papillary serous adenocarcinoma: 4				
			Adenocarcinoma, endocervical type: 4				
			Mullerian mixed tumor: 4				
			Carcinoma, NOS: 3				
			Clear cell adenocarcinoma, NOS: 3				
			Squamous cell carcinoma, keratinizing, NOS: 3				
			Papillary adenocarcinoma, NOS: 2				
			Mucinous cystadenocarcinoma, NOS: 1				
			Adenosquamous carcinoma: 1				
			Squamous cell carcinoma, spindle cell: 1				
			Mesodermal mixed tumor: 1				
			Serous surface papillary carcinoma: 1				
			Adenosarcoma: 1				
			Clear cell adenocarcinofibroma: 1				
			Papillary carcinoma, NOS: 1				
			Adenocarcinoma, intestinal type: 1				
Matched only (64)	62.5	White: 53	Endometrioid adenocarcinoma, NOS: 19	Grade 4/ Undifferentiated: 2 (3%)	IV: 16 (25%)	593	30
		Black/African American: 9	Serous cystadenocarcinoma, NOS: 7	Grade 3/ Poorly differentiated: 43 (67%)	III no IIIC: 13 (20%)		
		Hawaiian/Pacific Islander: 1	Carcinosarcoma, NOS: 6	Grade 2 / Moderately differentiated: 11 (17%)	IIIC: 9 (14%)		
		Other/Unknown: 1	Adenocarcinoma, NOS: 4	Grade 1 / Well differentiated: 4 (6%)	II: 6 (9%)		
			Mixed cell adenocarcinoma: 4	Unknown / Not determined: 3 (5%)	I: 17 (27%)		
			Squamous cell carcinoma, NOS: 4	None / Not applicable: 1 (2%)	Unknown: 3 (5%)		
			Mullerian mixed tumor: 3				
			Papillary serous cystadenocarcinoma: 2				
			Adenocarcinoma, endocervical type: 2				
			Papillary adenocarcinoma, NOS: 2				
			Clear cell adenocarcinoma, NOS: 2				
			Carcinoma, NOS: 2				
			Adenocarcinoma, intestinal type: 1				
			Papillary carcinoma, NOS: 1				
			Clear cell adenocarcinofibroma: 1				
			Serous surface papillary carcinoma: 1				
			Papillary serous adenocarcinoma: 1				
			Adenosquamous carcinoma: 1				
			Mucinous cystadenocarcinoma, ^NOS: 1^				
Unmatched (48)	63.0	White: 39	Endometrioid adenocarcinoma, NOS: 9	Grade 4/ Undifferentiated: 2 (4%)	IV: 12 (25%)	449	40
		Black/African American: 6	Squamous cell carcinoma, NOS: 7	Grade 3/ Poorly differentiated: 29 (61%)	III no IIIC: 9 (19%)		
		Asian: 2	Mixed cell adenocarcinoma: 6	Grade 2 / Moderately differentiated: 11 (23%)	IIIC: 7 (15%)		
		American Indian/Alaskan Native: 1	Adenocarcinoma, NOS: 5	Grade 1 / Well differentiated: 3 (6%)	II: 6 (12%)		
			Squamous cell carcinoma, keratinizing, NOS: 3	Unknown / Not determined: 3 (6%)	I: 14 (29%)		
			Papillary serous cystadenocarcinoma: 3				
			Papillary serous adenocarcinoma: 3				
			Carcinosarcoma, NOS: 2				
			Adenocarcinoma, endocervical type: 2				
			Serous cystadenocarcinoma, NOS: 2				
			Mullerian mixed tumor: 1				
			Adenosarcoma: 1				
			Clear cell adenocarcinoma, NOS: 1;				
			Mesodermal mixed tumor: 1				
			Carcinoma, NOS: 1				
			Squamous cell carcinoma, spindle cell: 1				

In Table 3, the number of patients treated with a drug is shown in the first column, and the number of continuous treatment periods is given in all other columns, i.e. treatments of the same patient with intervening periods are counted separately. The drugs given to the most number of patients were carboplatin and paclitaxel (93 patients each), and cisplatin (42). The most common drugs received overall as measured by separate treatments were also carboplatin, paclitaxel, and cisplatin, and these were given in similar proportions in the matched and unmatched treatments. Patients received a very similar number of treatments overall whether they were in the matched or unmatched groups i.e. 3.53 compared to 3.62 drugs.

**Table 3:** Drugs given most often for the matched and unmatched treatment groups compared against all patients, and also the most common drugs that were predicted to be beneficial, lacking benefit, or neither

Number of Patients Treated	Most Frequently Administered Drugs (Total Treatment Periods)							
All patients treated	All patients – treatment periods	Matched only patients, all treatments	Matched, after profiling treatments only	Unmatched patients, all treatments	Unmatched, after profiling treatments only	Drugs predicted of benefit	Drugs predicted to lack benefit	Drugs with no prediction (neither of benefit or lack of benefit)
carboplatin; paclitaxel – 93 patients	carboplatin (112)	carboplatin; paclitaxel (62)	carboplatin (34)	carboplatin (50)	carboplatin (23)	carboplatin (51)	carboplatin (31)	paclitaxel (61)
-	paclitaxel (110)	-	paclitaxel (33)	paclitaxel (48)	paclitaxel (20)	cisplatin (23)	paclitaxel (23)	carboplatin (28)
cisplatin – 42 patients	cisplatin (48)	cisplatin (23)	cisplatin (14)	cisplatin (25)	cisplatin (8)	paclitaxel (22)	cisplatin (18)	bevacizumab (9)
doxorubicin hydrochloride – 18 patients	doxorubicin hydrochloride (20)	doxorubicin hydrochloride (12)	doxorubicin hydrochloride (7)	gemcitabine hydrochloride (10)	gemcitabine hydrochloride (7)	doxorubicin hydrochloride (13)	doxorubicin hydrochloride (7)	docetaxel (8)
bevacizumab; gemcitabine hydrochloride – 11 patients	bevacizumab; gemcitabine hydrochloride (14)	docetaxel (9)	bevacizumab (5)	bevacizumab; doxorubicin hydrochloride (8)	bevacizumab; doxorubicin hydrochloride (4)	pegylated liposomal doxorubicin hydrochloride (11)	gemcitabine hydrochloride (4)	cisplatin (7)
-	-	pegylated liposomal doxorubicin hydrochloride (7)	megestrol acetate; docetaxel (4)	-	-	gemcitabine hydrochloride (10)	letrozole; pemetrexed disodium; anastrozole (2)	patupilone (5)
pegylated liposomal doxorubicin hydrochloride – 10 patients	pegylated liposomal doxorubicin hydrochloride (11)	bevacizumab; topotecan hydrochloride (6)	-	pemetrexed disodium; pegylated liposomal doxorubicin hydrochloride (4)	pegylated liposomal doxorubicin hydrochloride; pemetrexed disodium (3)	tamoxifen citrate (6)	-	ifosfamide; topotecan hydrochloride (4)
docetaxel; topotecan hydrochloride – 9 patients	docetaxel (10)	-	pegylated liposomal doxorubicin hydrochloride; topotecan hydrochloride (3)	-	-	megestrol acetate (4)	-	dalantercept; vinorelbine tartrate; nab-paclitaxel; capecitabine; temsirolimus (2)
-	topotecan hydrochloride (9)	tamoxifen citrate; megestrol acetate (5)	-	topotecan hydrochloride (3)	topotecan hydrochloride (2)	bevacizumab; pemetrexed disodium (3)	oxaliplatin; docetaxel; irinotecan hydrochloride; topotecan hydrochloride; temozolomide (1)	-
tamoxifen citrate – 6 patients	tamoxifen citrate (6)	-	-	dalantercept (2)	docetaxel; temsirolimus; dacarbazine; vinorelbine tartrate; ifosfamide (1)	-	-	-

The most commonly given drugs are listed in descending order (the number of treatments is given in parentheses).

Including the time prior to profiling, in the matched group 53% of drugs given (120 treatments) were predicted to be beneficial, 4% (9 treatments) lacked benefit, and 43% (97) were neither of these. In the unmatched 21% (37 treatments) were profiled as beneficent, 49% (85) lacked benefit, and 30% (52) being neither. 38% of patients in the unmatched subset received at least one drug proposed to be of benefit, and 19% received two or more beneficial drugs.

Overall carboplatin, paclitaxel and cisplatin were the most frequently selected cytotoxic drugs, and interestingly platinums were given frequently in both the matched and unmatched category; 45% of treatments with carboplatin were given to patients who were predicted not to benefit from the agent. When considering only treatments after the time of tumor profiling, these drugs were still the most often administered drugs in both the matched and unmatched groups. Some drugs that were used had no recommendation associated with them, and were placed in a neither (or neutral) category. The most common drug in the neutral category was paclitaxel (given for 61 time periods, i.e. 15% of all treatments overall for this cohort).

### Biomarkers explored

**Figure 2:**
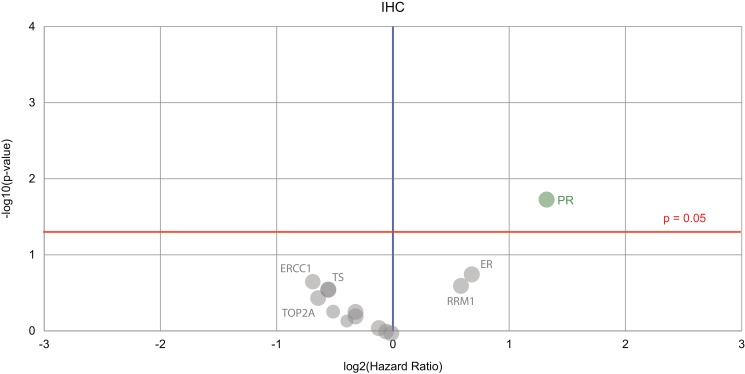
Volcano plot of biomarkers showing prognostic value for female genital tract cancer survival The biomarker of significance–found on the right in green–is the IHC marker for the progesterone receptor (PR). Green circle = the hazard rate of a positive biomarker result is significantly lower than that of a negative biomarker result, gray circles = the difference between a positive biomarker result and a negative biomarker result is not significant.

The only baseline biomarker predictive of better outlook and improved overall survival was the PR overexpression (Figure 2). Differences in the IHC biomarkers between the matched and unmatched groups are also compared in Figure 1B.

### Matched treatments compared to unmatched–characteristics and outcomes

The data was divided into two groups; one whose treatments always matched those recommended using their biomarker profiles and in the other they did not. In the matched treatment group there were 64 patients, and these all received one or more recommended drugs after the time of collection for molecular profiling and concurrently none that were predicted to lack benefit after sample collection. In the unmatched treatment group there were 48 patients, and these were all given at least one drug that was expected to lack benefit after collection for profiling.

**Figure 3:**
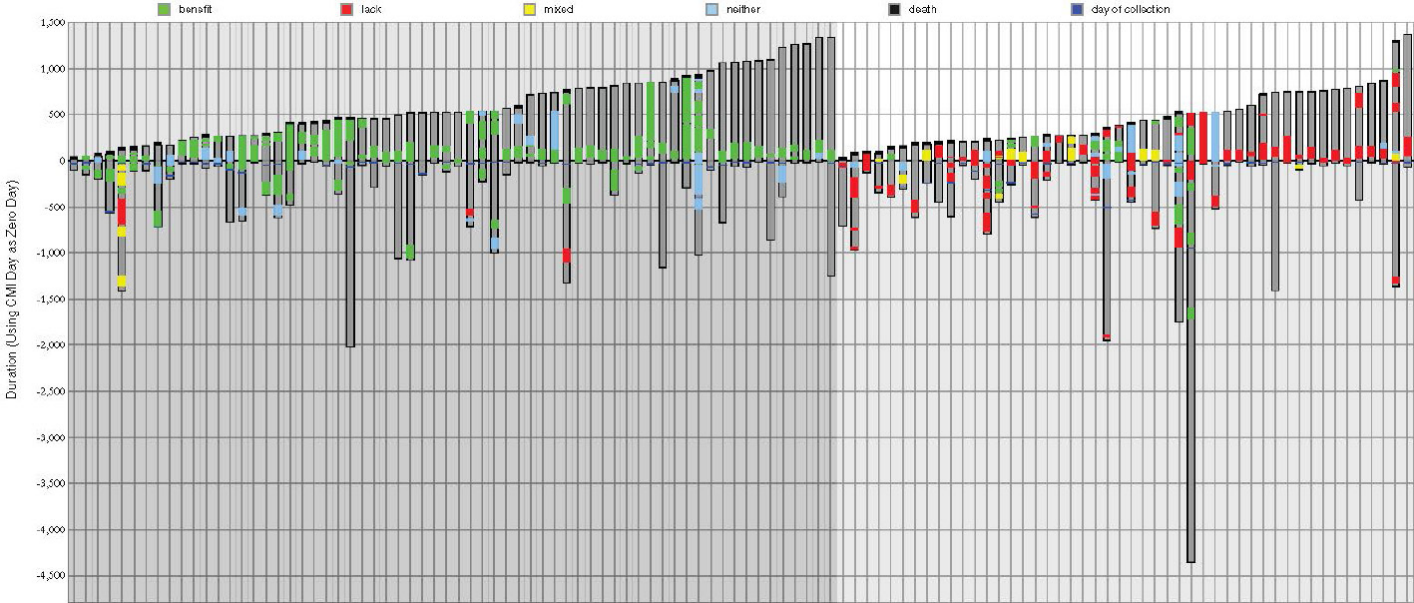
Treatments ordered by survival time for matched and unmatched patients Regimens followed by 64 matched patients in ascending survival time (after profiling) are on the left (with a darker gray background), and treatments for 48 unmatched patients are on the right (light gray background), also ordered by survival time. Each column corresponds to an individual patient. The ordinate is time (days) where zero is when molecular profiling of the patient’s tumor occurred. If a black line is present at the top of a bar this indicates death. Dark gray within a bar shows the time from diagnosis to either last time of monitoring or death. Green is a period while receiving a drug of benefit. Red is a drug designated by Caris as lacking benefit. Yellow is a combination of treatments that are both of benefit and lack thereof. Blue is a neutral therapy, i.e. neither benefit nor lack of benefit.

The survivals of the two groups are compared in waterfall plots in Figure 3, where each bar displays a treatment plan for an individual cancer patient. The 112 bars in total represent the 64 matched (on the left with a darker grey background) and 48 unmatched patients (on the right with a lighter grey background), and each is ordered from left to right by survival time after profiling was performed, so that post-profiling survival time increases across the plot. Drugs predicted to be of benefit and those that lack benefit are indicated with color coded bands–green, red and yellow indicate drugs of benefit, lack of benefit and neither of these, respectively.

Patients in the matched group on average survived for 593 days after profiling, compared to 449 days for patients from the unmatched group; this is an increase of 32% (*P =* 0.0265). In the matched group 30% of patients were deceased when monitoring finished, compared to 40% of the unmatched group patients. Figure 1A also shows a Kaplan-Meier curve, where there is an increase in overall survival for the patients that were only treated with drugs expected to be beneficial.

The patients who received one or more drugs predicted to lack benefit had a worse overall survival (OS) in general than the patients who received a single drug that were in this category. If patients with endometrioid adenocarcinoma are removed, the average survival after monitoring is 555 days versus 431 days for matched versus unmatched (*P =* 0.0797); this might be because they had fewer treatment options. If instead the patients that received megestrol acetate or tamoxifen citrate are removed, survival changes to 610 days for matched patients and 447 days for the unmatched group (*P =* 0.0176).

## DISCUSSION

This report analyzed a female genital tract malignancy cohort, that received treatment recommendations based on molecular profiling of their tumors, by Caris Life Sciences using mostly immunohistochemistry (IHC) indicators. Patients whose treatments concurred with their recommendations, were compared to those that did not because they received at least one drug that was designated by Caris as lacking benefit. The group of patients that matched profiling recommendations had an increase of 32% in survival compared to the unmatched set of patients, which is an increase of 144 days, from 449 to 593 days.

The drugs given most often for all patients were carboplatin (112 times), paclitaxel (110 times) and cisplatin (48 times). This was also true in the matched and unmatched subsets, whether including all treatments or only those after the time of profiling.

The matched and unmatched groups received similar numbers of treatments (3.53 vs 3.62). However, the unmatched subset was comprised of patients with shorter survival times and higher mortality rates. This may have been influenced by the unmatched group having tumors that were generally more advanced than those in the group of matched patients, as shown in Table 2. We further hypothesize that the overexpression of ERs and PRs in endometrial cancers in particular, has contributed to the survival difference seen in the matched versus unmatched cohorts. Of the 112 patients, 10 received megestrol or tamoxifen, and response to ER/PR directed therapies is likely to have improved survival in this matched cohort. This is perhaps reflected in the PR status being the only IHC biomarker that was significantly associated with better survival (Figure 2). In other cohorts, gynaecological cancers such as endometrial cancer or low grade ovarian cancers that overexpress ERs and PRs can respond to hormonal therapies [7, 9], and it has been noted that PR status indicates better prognosis in ovarian and endometrial cancers [10–13], although in cervical cancer the receptor status is not thought to be correlated with survival [14].

Although we have only identified PR as standing out from the other biomarkers in terms of prognostic ability, this does not mean that other markers used within the panels do not contribute in a cumulative fashion to influence accuracy of predictions in a more subtle but important way, for example, the other markers that are labelled in the IHC volcano plot shown in Figure 2.

This is likely to account for much of the difference in survival between the two cohorts. Despite this, different cytotoxic agents such as pemetrexed and capecitabine were used with benefit, as was the mTOR inhibitor temsirolimus in the matched cohort. This data shows that there is differential expression of potentially interesting biomarkers such as *ERCC1*, *TS* and *PTEN* in gynaecological cancers. However, no conclusions are possible regarding their effect on drug response given the small dataset.

Overall, although tumor molecular profiling is in its infancy, we find that molecular profiling results can be used successfully to improve treatment of female genital tract cancer, but that this is largely as a result of identification of established IHC predictors of response to hormonal therapy. The benefit of molecular profiling in gynaecological malignancies will likely continue to grow significantly as treatments of these tumors are increasingly utilizing targeted strategies such as anti-angiogenics, PARP inhibition, and immune modulation. More data is needed to validate in prospective studies the application of IHC and sequencing-based prognostic and predictive biomarkers, in gynaecological cancers.

## MATERIALS AND METHODS

The Caris CODE database (Comprehensive Oncology Database Explorer) version 1.0 contains tumor molecular profile data for 841 patients with solid tumors. It contains demographic information about these patients, the drug treatments that they received before and after molecular profiling and their clinical outcomes. There are 112 advanced stage female genital tract cancer patients described within this resource, and we mined this cohort after web scraping the data, to assess how much tumor profiling recommendations were used in drug selection by clinicians, and if any molecular subsets had different outcomes. Tables 1 and 2 describe the clinical and demographic characteristics of the female genital tract cohort that was studied here.

The amount of time that patients were monitored varied, as shown in Figure 3; on average patients’ treatment records were available for 921 days after diagnosis (938 for matched treatment patients, 897 for unmatched), and on average the time of monitoring after profiling was 531 days. The longest amount of time that treatment records were available, i.e. before and after profiling up until the last day of contact, was 4871 days. The longest time of monitoring after profiling (the patient represented on the furthest right of Figure 1) was 1366 days which was 1440 days after diagnosis.

## Abbreviations

*ER*: *estrogen receptor*
*IHC*: *immunohistochemistry*
*OS*: *overall survival*
*PR*: *progesterone receptor*
