# Correction: Assessing tumor molecular profiling to guide treatments for patients with advanced female genital tract malignancy

**DOI:** 10.18632/oncotarget.24689

**Published:** 2018-03-13

**Authors:** Philip Carter, Costi Alifrangis, Biancastella Cereser, Pramodh Chandrasinghe, Lisa Del Bel Belluz, Christina Fotopoulou, Andreja Frilling, Thomas Herzog, Nina Moderau, Neha Tabassum, Jonathan Krell, Justin Stebbing

**Affiliations:** ^1^ Department of Surgery and Cancer, Imperial College, London, UK; ^2^ Department of Oncology, University College Hospital, London, UK; ^3^ Department of Surgery, University of Kelaniya, Kelaniya, Sri Lanka; ^4^ Department of Obstetrics and Gynecology, University of Cincinnati, Cincinnati, USA; ^5^ University of Cincinnati Cancer Institute, University of Cincinnati, Cincinnati, USA

**This article has been corrected:** The proper Materials and Methods and Conflicts of Interest information is as follows:

**MATERIALS AND METHODS**

The Caris CODE database (Comprehensive Oncology Database Explorer) version 1.0 contains tumor molecular profile data for 841 patients with solid tumors. It contains demographic information about these patients, the drug treatments that they received before and after molecular profiling and their clinical outcomes. There are 112 advanced stage female genital tract cancer patients described within this resource, and we mined this cohort after web scraping the data, to assess how much tumor profiling recommendations were used in drug selection by clinicians, and if any molecular subsets had different outcomes. Tables 1 and 2 describe the clinical and demographic characteristics of the female genital tract cohort that was studied here.

The amount of time that patients were monitored varied, as shown in Figure 3; on average patients’ treatment records were available for 921 days after diagnosis (938 for matched treatment patients, 897 for unmatched), and on average the time of monitoring after profiling was 531 days. The longest amount of time that treatment records were available, i.e. before and after profiling up until the last day of contact, was 4871 days. The longest time of monitoring after profiling (the patient represented on the furthest right of Figure 1) was 1366 days which was 1440 days after diagnosis.

The data were analysed independently of Caris. Patients were covered under 1 of 4 different protocols or exemptions, listed as follows. (1). The Caris Registry Protocol (TCREG-001-00-V2-1209) was approved by WIRB (WIRB Tracking #20092285) and has an NCT# of NCT02678754. (2). The Caris POA Prospective Repository (COE-001-0815) was approved by WIRB (WIRB Tracking #20162864) and has an NCT# of NCT03324841. (3). The Caris POA Retrospective Repository (COE-002-0116) was approved by WIRB (WIRB Tracking #20162657) and has an NCT# of NCT 00326499. (4). ION data is covered under an IRB exemption. All data are retrospective and have been de-identified prior to Caris receiving it and authors performing independent analyses.

**CONFLICTS OF INTEREST**

Authors received no funding or honoraria for this publication. The data were analysed independently of Caris. Thomas Herzog is on the scientific advisory board of Caris Life Sciences.

Original article: Oncotarget. 2018; 9:6007-6014. https://doi.org/10.18632/oncotarget.23675

